# Preliminary Effectiveness of a Postnatal mHealth and Virtual Social Support Intervention on Newborn and Infant Health and Feeding Practices in Punjab, India: Quasi-Experimental Pre-Post Pilot Study

**DOI:** 10.2196/65581

**Published:** 2025-06-27

**Authors:** Garima Singh Verma, Lakshmi Gopalakrishnan, Alison El Ayadi, Nadia Diamond-Smith, Rashmi Bagga, Shashi Kant Dhir, Pushpendra Singh, Navneet Gill, Vaibhav Miglani, Naveen Mutyala, Ankita Kankaria, Jasmeet Kaur, Alka Ahuja, Vijay Kumar, Mona Duggal

**Affiliations:** 1 Department of Obstetrics and Gynecology, Postgraduate Institute of Medical Education and Research, Chandigarh, India; 2 Institute for Global Health Sciences, University of California San Francisco, San Francisco, CA, United States; 3 Bixby Center for Global Reproductive Health, Department of Obstetrics, Gynecology, and Reproductive Sciences, University of California San Francisco, San Francisco, CA, United States; 4 Department of Epidemiology and Biostatistics, University of California San Francisco, San Francisco, CA, United States; 5 Department of Pediatrics, Guru Gobind Singh Medical College and Hospital, Faridkot, India; 6 Department of Computer Science and Engineering, Indraprastha Institute of Information Technology, Delhi, India; 7 Department of Hematology, Postgraduate Institute of Medical Education and Research, Chandigarh, India; 8 Department of Community Medicine and School of Public Health, Postgraduate Institute of Medical Education and Research, Chandigarh, India; 9 SWACH – Foundation for Survival of Women and Children, Panchkula, India; 10 National Institute for Research in Digital Health and Data Science, Indian Council of Medical Research, Ansari Nagar East, New Delhi, 110029, India

**Keywords:** digital health, mHealth, mobile health, app, postpartum, health education, social support, India, infant morbidity, care seeking, postnatal, maternal, mother, text message, virtual support, online support, pediatric, infant, baby, neonate, newborn, breastfeeding, feeding

## Abstract

**Background:**

We evaluated a pilot mobile health (mHealth) intervention aimed at improving postnatal maternal and infant health. The intervention featured provider-led group sessions for education, health care communication, in-person care referrals, and virtual mHealth support for postpartum mothers through weekly calls, texts, interactive voice response (IVR), and a phone app.

**Objective:**

We aimed to assess the preliminary effectiveness of the pilot mHealth intervention, *MeSSSSage* (*Maa Shishu Swasthya Sahayak Samooh*, which means maternal and child health support group), on infant health knowledge, behaviors, and outcomes at 6 months post partum. We focus on maternal knowledge of infant danger signs and optimal young child feeding practices at 6 months post partum and also evaluate maternal care-seeking behaviors for infants, adherence to age-appropriate immunization, and infant and young child feeding practices such as early initiation of breastfeeding and complementary feeding.

**Methods:**

We evaluated the preliminary effectiveness of an intervention on maternal health knowledge among 135 participants in Punjab, India, who completed pre- and postintervention surveys. The intervention, led by research personnel with backgrounds similar to community health officers, aimed to empower society and support universal health coverage if successful. We assessed changes in knowledge of maternal danger signs and the appropriate age for introducing different food groups over 6 months post partum. Additionally, we examined postintervention differences in health-seeking behavior for infants, adherence to age-appropriate immunizations, and adoption of breastfeeding and complementary feeding practices among women in the synchronous (group call), asynchronous (IVR and app), and control arms.

**Results:**

Of 12 infant risk factors, maternal knowledge of infant danger signs remained low (mean range: 1.85-2.31 preintervention and 1.81-2.22 postintervention). Participants in the synchronous arm had a statistically significant higher mean increase (mean difference: 0.87, 95% CI 0.06‐1.69) compared to the control arm. Participants in synchronous arms had nearly 3-fold increased odds of infant health checkup by a clinical provider than asynchronous arm participants (odds ratio [OR] 2.72, 95% CI 1.02-7.23). No significant differences were noted in age-appropriate vaccine coverage among infants between arms, though vaccination coverage was more than 80% across all arms. Early initiation of breastfeeding remained low across all arms (~47%).

**Conclusions:**

Our pilot study on group-based mHealth education and virtual social support during the postnatal phase showed modest yet promising results. Rigorous testing is crucial to strengthening the limited evidence base for group-oriented mHealth approaches.

## Introduction

### Background

India has made substantial improvements in child survival through a 54% reduction in neonatal mortality rate from 52 per 1000 live births in 1990 to 23.7 per 1000 live births in 2017 [1,2]. About 75% of newborn deaths occur in the first week of life, highlighting the importance of intervening in the early postnatal period [3]. Further, child undernutrition accounts for over 20% of deaths and remains a major risk factor for disease burden in children younger than 5 years in India [2,4]. The latest national survey suggests that 35.5% of children in India are stunted, 32.1% are underweight, and 19.3% are wasted with substantial variation both across and within Indian states [5].

There is a robust evidence base of interventions spanning the continuum of care from pregnancy through post partum and through the first 1000 days of life, including appropriate infant and young child feeding practices to improve newborn, and child health and nutrition outcomes [6,7]. India, backed by a two million+ female community health worker program, has adopted a comprehensive and universally available package of maternal and neonatal evidence-based interventions through its multiple national government programs, including the Integrated Child Development Services, the National Health Mission, and the National Nutrition Mission [8,9]. However, national surveys suggest that coverage of nutrition interventions, particularly in the postnatal and newborn period, remains low. Only 41.8% of children were breastfed within an hour of birth, 46% of children 6‐8 months were initiated on complementary food, and 11% of children below 6‐23 months received adequate diet with disparities by geographical region, socioeconomic status, and rural-urban residence [5].

Prior research from India suggests that postnatal care is associated with reduced neonatal mortality [10], and appropriate care for sick infants and children is correlated with reduced risk of severe wasting [11]. Postnatal education is an evidence-based strategy to improve newborn health and nutrition and maternal knowledge of general infant health and care [12]. While national data suggest that 79% of infants receive a postnatal checkup by skilled health personnel within 2 days of childbirth [5], a recent study of 13,000+ respondents from 3 large Indian states (including Punjab, one of our study states) highlights that 55% of mothers receive no postnatal education, with considerable knowledge gaps in appropriate newborn care practices, including skin-to-skin care, cord care, warning signs of infant illness, among other related topics [13]. Beyond postnatal education, enhancing social support for mothers through interpersonal connections is associated with reduced risk of postpartum depression [14,15], improved maternal self-efficacy [16,17], higher postnatal care attendance of mothers and infants [18], increased healthy maternal physical activity and nutritional intake [14], and better infant and young child feeding practices [19]. However, various barriers hinder postnatal care in India, including logistical challenges exacerbated by geographic distance and cultural factors [20-22]. Factors such as lower levels of women’s autonomy, restricted freedom of movement, and social isolation further compound these challenges, particularly during the postnatal period when women are traditionally confined to their homes for 40 days after delivery [23]. Additional barriers include poverty, limited education, lack of male involvement, absence of health insurance, financial constraints, and perceptions of inadequate quality or lack of benefit of services [24-26].

Leveraging mobile health (mHealth)–based interventions holds promise in addressing barriers to postnatal care and improving postnatal maternal knowledge, social connectedness, and maternal and child health-related behaviors given increasing mobile phone ownership and internet access. A scoping review of 28 studies across low- and middle-income countries (LMICs) found improved immunization coverage and increased adherence to immunization schedules through mHealth-based text and voice message reminders [27], while another systematic review of 16 studies in LMICs found mHealth education interventions to increase perinatal interactions between young mothers and health care workers [28]. Individual studies reporting on mHealth interventions using text and voice messaging have improved perinatal care attendance [29], early initiation of breastfeeding and exclusive breastfeeding [30-32], protein consumption [33], general infant and young child feeding practices [34], knowledge of child immunization [35], and better adherence to child immunization schedules [36].

Key gaps in the evidence base on the impact of mHealth interventions on postpartum and neonatal health in India include fewer interventions focused on this topic area, with most targeting frontline health and nutrition workers to improve service delivery [37-40], limited interactions and social support provision among those targeting beneficiaries [30-32,41], and the need for more rigorous studies [28,30,37,40]. Group-oriented mHealth interventions combining group-based learning and social support may overcome prevalent barriers given the convenience, feasibility, acceptability, and preliminary effectiveness in improving postnatal maternal and infant health outcomes [32,42-47]. However, the combined impact of interactive group-based social support delivered via mHealth modalities such as group voice calls or Zoom has been understudied in LMICs.

**Figure 1.**
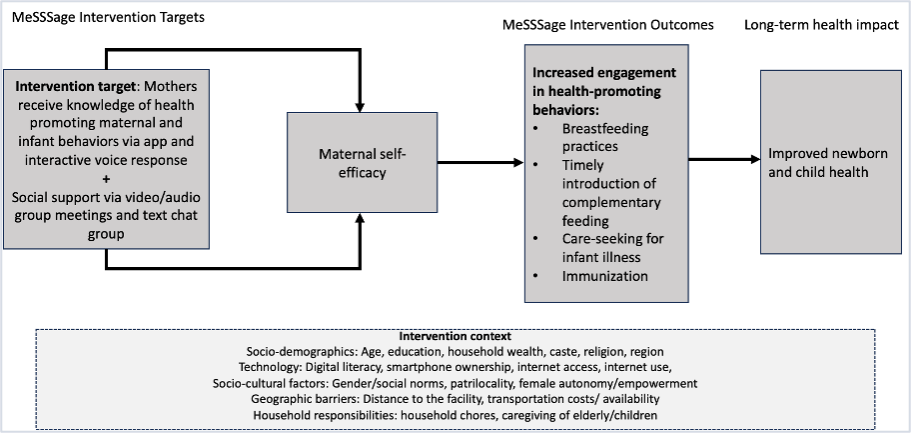
Conceptual framework of intervention context, *MeSSSSage* intervention targets and outcomes, and anticipated long-term impacts. *MeSSSSage*: *Maa Shishu Swasthya Sahayak Samooh* (which means maternal and child health support group).

To address these gaps, we designed a provider-facilitated group perinatal mHealth intervention called *MeSSSSage* (*Maa Shishu Swasthya Sahayak Samooh*, which means maternal and child health support group). Informed by the capabilities and motivation constructs of the COM-B (Capability, Opportunity, and Motivation Behavior) framework [48,49], the intervention was designed to enhance maternal knowledge of health-promoting behaviors and promote maternal self-efficacy and empowerment to improve health-related knowledge, behaviors, and maternal and child outcomes (Figure 1). The intervention aimed to improve knowledge, facilitate referrals to in-person care, and foster connections with a virtual social support group for postnatal mothers with infants of similar ages through various mHealth modalities, including weekly group calls and text chat. Our development process included 2 iterative rounds of pilot testing—the first to inform the mHealth components and design factors [50] and the second to understand the feasibility and acceptability of our revised intervention [51] and explore preliminary effectiveness on health outcomes.

### Objective of This Study

In the current analysis, we aimed to assess the preliminary effectiveness of the pilot mHealth intervention, *MeSSSSage*, on infant health knowledge, behaviors, and outcomes at 6 months post partum. We focus on maternal knowledge of infant danger signs and optimal young child feeding practices at 6 months post partum and also evaluate maternal care-seeking behaviors for infants, adherence to age-appropriate immunization, and infant and young child feeding practices such as early initiation of breastfeeding and complementary feeding.

### *MeSSSSage* Intervention

**Figure 2.**
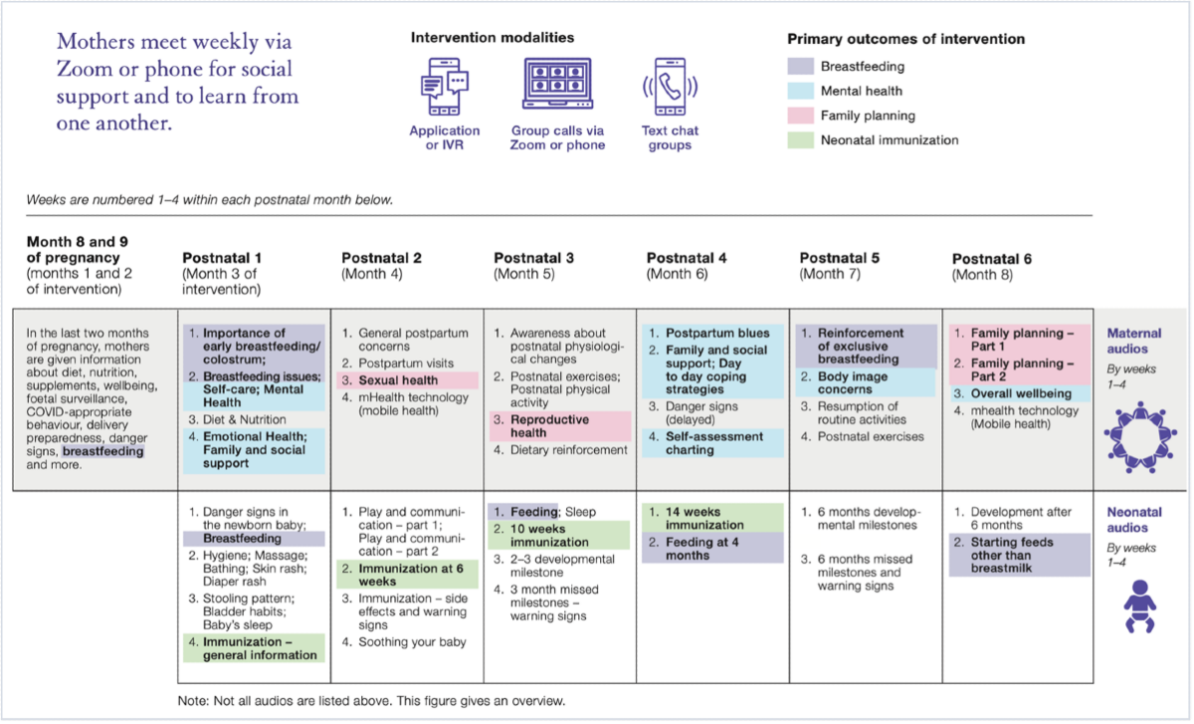
Weekly maternal and neonatal content of *MeSSSSage* intervention. IVR: interactive voice response; *MeSSSSage*: *Maa Shishu Swasthya Sahayak Samooh* (which means maternal and child health support group).

The *MeSSSSage* intervention was administered to women from late pregnancy through 6 months post partum. It included weekly audio or audio-video group calls, group text chats, and audio educational content provided via automated interactive voice response (IVR) or *MeSSSSage* app (Table 1 and Figure 2). Groups and group-based content (ie, audio-video group calls and group text chats) were facilitated by trained intervention moderators with backgrounds in public health or social work and supported by a gynecologist and neonatologist. The detailed description is published elsewhere [51].

**Table 1.** *MeSSSSage^a^* intervention modalities.

mHealth^b^ modalities or arms	Description
Audio-video group sessions	Trained moderators led weekly group sessions focusing on increasing education and social support. These sessions incorporated icebreakers and group-building activities, facilitated discussions based on weekly themes (Figure 2), and allowed open questions and discussion sessions. Prenatally, a gynecologist participated in one call per month, while postnatally, both a gynecologist and a neonatologist participated in one call per month. Participants could choose to connect to their group via audio-only sessions on the TATA platform or video sessions on the Zoom platform.
WhatsApp-based group text chat	Trained moderators led weekly WhatsApp-based group chats by sharing audio and visual messages based on themes (Figure 2). Group participants were expected to engage by asking questions, and group discussion was facilitated.
*MeSSSSage* mobile app	Weekly educational audio messages focus on key information regarding weekly themes (Figure 2). The mobile app structured sections for these weekly audio messages, offering women the flexibility to access health education content at their convenience.
IVR^c^	IVR calls were scheduled to reach participants once a week at designated days and times. To maximize the chances of participants receiving the calls, they were sent out 3 times within a 15-minute interval. These brief audio calls, lasting between 5 and 10 minutes each, addressed essential topics concerning perinatal, neonatal, and child health. IVR calls persisted up to 6 months postdelivery.

a *MeSSSSage*: *Maa Shishu Swasthya Sahayak Samooh* (which means maternal and child health support group).

b mHealth: mobile health.

c IVR: interactive voice response.

Figure 2 provides a summary of the intervention plan. While this table offers the general structure of IVR calls and audio-video group discussion topics, the audio-video group discussion topics were dynamically adjusted based on the queries and concerns shared by the new mothers, ensuring a responsive and participant-focused approach.

## Methods

### Study Design

This open-label pilot study on an mHealth-based perinatal health support intervention targeted women in late pregnancy through 6 months post partum in the Boothgarh block of the state of Punjab, northern India. We used a pretest-posttest nonrandomized control group design. Quantitative survey data were collected at study enrollment and intervention completion (approximately 6 months post partum).

### Study Participants

Participants were eligible for inclusion in the study if they met the following criteria: (1) aged 18 years or older, (2) between 28 and 32 weeks pregnant, (3) residing in the study area, and (4) not experiencing a serious maternal complication. Participants with high-risk pregnancies and parity of more than 2 were ineligible.

### Study Procedures

Our study team used antenatal clinic registry data maintained by community health workers to prescreen pregnant women in the seventh month of gestation. We then contacted these potential participants over the phone, screened them, and if found eligible, invited them to participate. We led them through an informed consent process, which included discussing study procedures, risks, and benefits. All participants provided informed consent verbally.

Out of the 397 women we tried to reach, we successfully recruited a total of 180 participants. These participants were then sequentially assigned to one of five arms: (1) app only (n=20), (2) IVR only (n=20), (3) group call+WhatsApp+app (3 separate arms, n=60), (4) group call+WhatsApp+IVR (3 separate arms, n=60), and (5) finally the control arm (n=20) at baseline. All participants, including the control arm, received the standard of care, which, in this setting, comprised community health worker–led home visits, counseling, and immunization services. Reasons for nonenrollment included being unable to reach the potential participant over the phone, as their phone was either switched off or out of service. Some women provided false information, while others were no longer pregnant due to a miscarriage or preterm birth. A few participants also described having irregular access to mobile phones since they used either their husband’s or an immediate family member’s phone and only had access to it in the evening when their husband or family member returned home from work. Some individuals did not respond to our calls, while others faced language challenges.

After enrollment, the 8 groups or arms were formed in a staggered fashion, leading to a study timeline from August 2021 to November 2022. Participant engagement in the intervention spanned a total of 8 months. Enrollment of participants and administration of the baseline survey occurred between August and December 2021. Intervention implementation spanned from August 2021 to July 2022, and our endline quantitative survey was conducted between May and December 2022. All data were collected through interviewer administration over the phone and directly entered into the REDCap (Research Electronic Data Capture) data collection application. For individuals who were not able to be reached for the endline survey, local ASHAs (Accredited Social Health Activists) were engaged to facilitate in-person quantitative survey administration.

### Study Measures

Based on existing research on IVR and apps for maternal and child health in India, we focused on examining the supplementary impact of group calls. We anticipated that participants engaged in group calls would experience higher levels of social connectedness compared to those using the educational app or IVR, as the former allowed for direct interaction with others, fostering a sense of community and support. For analysis, we consolidated study participants into 3 intervention categories: a synchronous arm (combining arms 3 and 4 described above, comprising all group call participants), an asynchronous arm (combining arms 1 and 2 described above, comprising those in the app and IVR), and a control arm.

The primary outcomes of this analysis were the change in maternal knowledge of infant danger signs and maternal knowledge of infant and young child feeding practices between pre- and postintervention. Knowledge of infant danger signs was defined as women’s ability to recall symptoms indicating infants may require medical attention within the first month of birth. We developed a cumulative score based on women’s recall of various danger signs, including diarrhea, fever, cough or cold, difficulty breathing, absence of crying, chest problems, blue tongue and lips, lack of milk intake, failure to gain weight, premature birth, jaundice, coldness to touch, and others (scores ranging from 0 to 13). Women’s knowledge of infant and young child feeding practices was assessed by their correct reporting of the appropriate age for introducing the following foods: water, rice, bread, legumes (dal), green leafy vegetables, pumpkin, carrot, fruits (banana, papaya, mango, and orange), meats (chicken, mutton, and fish), eggs, and different types of milk (cow, goat, powdered, etc). Women who correctly identified 6 months or older as the appropriate age for introducing all food groups except cow’s/goat’s milk (12 months) were coded as 1; otherwise, they were coded as 0. We developed a cumulative score based on women’s correct responses.

Additional outcomes assessed postintervention included infant health status, access to health care, infant vaccination status, and infant feeding behaviors. Infant postnatal health characteristics were assessed through various questions. Postnatal care was assessed based on women’s self-reports regarding whether their infants received a checkup within 6 weeks of birth, the frequency of these checkups, and the type of provider conducting the checkup (clinical or community). Infant vaccination coverage was determined by whether the infant had received all age-appropriate vaccines. Infant and young child feeding practices were evaluated through breastfeeding, early initiation of breastfeeding (defined as within 1 hour of birth), and complementary feeding (defined as whether other liquids or foods had been introduced to the infant). All variables were coded as indicator variables (yes/no).

Participant sociodemographic characteristics collected during preintervention included age, relationship status, educational attainment, religion, caste, ration card and type, parity, and mobile phone ownership.

### Analysis

At baseline, 120 participants were in the synchronous arm, 40 participants in the asynchronous arm, and 20 in the control arm. At endline, we had 94 participants (78.3% retention rate) in the synchronous arm, 28 participants (70% retention rate) in the asynchronous arm, and 13 (65%) in the control arm. With our primary focus on temporal change, we restricted the analytic sample for this paper to participants with both baseline and endline data, resulting in a sample size of 135 participants.

We compared the sociodemographic characteristics of the 3 analysis arms (synchronous, asynchronous, and control arm) by identifying standardized differences [52]. Due to significant disparities across arms in the distribution of age, age at marriage, household composition, educational attainment, household income, ration card possession, mobile phone ownership, and smartphone access, we used inverse probability weighting to ensure comparability of participants across arms. This approach, similar to direct standardization, balanced preintervention discrepancies in sociodemographics between the arms [53,54].

We summarized sociodemographic characteristics using proportions and means of the matched, reweighted study population surveyed pre- and postintervention stratified by 3 arms (synchronous, asynchronous, and control). We then assessed the association of being in each intervention arm on primary outcomes (changes in maternal knowledge of infant danger signs and knowledge of infant and young child feeding practices) using mixed effects linear regression, including a random intercept for participants with robust standard errors to adjust within individual clustering due to the longitudinal structure of the data. The difference-in-difference coefficient (β) is the interaction term between a categorical variable denoting the time (before vs after the intervention was implemented) and the intervention arm (synchronous vs asynchronous modes, synchronous vs control, and asynchronous vs control). We interpreted this term as the differential change over time associated with being in each intervention arm compared to the reference group. For outcomes collected only at endline, we analyzed the differences between the arms (synchronous vs asynchronous modes, synchronous vs control, and asynchronous vs control) using logistic regression. Differences where *P*<.05 were considered statistically significant. All analyses are presented using weighted estimates. Data entry was done through REDCap, and all statistical analyses were conducted using Stata 15 (StataCorp LLC).

### Ethical Considerations

This study received approval from the Indian Council of Medical Research and senior health authorities of the Government of Punjab and the Mission Director, National Health Mission, India. The study protocol was approved by the University of California San Francisco Institutional Review Board (19‐299723), the ethics committee of the Postgraduate Institute of Medical Education and Research (IEC-03/2020‐1567), the Collaborative Research Committee of the Postgraduate Institute of Medical Education and Research (79/30-Edu-13/1089‐90), and the Indian Council of Medical Research (ID 2020‐9576). All study participants were deidentified, engaged in a thorough informed consent process and provided written confirmation of informed consent at enrollment.

## Results

### Sociodemographic Characteristics

At study enrollment, participants had an average age of 26.8 years, and almost all (99.3%) were married (Table 2). The majority had either a high school education (44.8%) or higher education (44.3%). Nearly two-thirds of the sample belonged to the Sikh religion (65.3%), and one-third of the sample belonged to a marginalized caste (scheduled caste and scheduled tribe; 36.4%). Less than half possessed a ration card (48%), an official government document given to eligible poor families to get subsidized food grains from government fair price shops. Parity was one (53.3%) or more (46.8%). Mobile phone ownership at the household level was near-universal (99.2%), and most women owned their own phones (92.5%).

**Table 2.** Sociodemographic characteristics of the intervention participants.

	Synchronous (n=94)	Asynchronous (n=28)	Control (n=13)	Total (N=135)
Age, mean (SD)	26.7 (0.38)	26.8 (0.61)	26.7 (1.57)	26.7 (0.33)
Relationship status, n (%)				
Married or domestic partnership	94 (100)	27 (98)	13 (100)	134 (99.8)
Separated	0 (0)	1 (1.2)	0 (0)	1 (0.2)
Educational attainment, n (%)				
None	0 (0)	2 (7.2)	1 (6)	3 (1.5)
Up to secondary	9 (9.6)	1 (3.1)	3 (22.2)	13 (9.4)
Higher secondary	41 (43.6)	15 (50.1)	6 (47.1)	62 (44.8)
Diploma or higher	44 (46.8)	10 (39.6)	3 (24.7)	57 (44.3)
Religion, n (%)				
Hindu	23 (24.5)	11 (32.2)	4 (26.3)	38 (25.7)
Muslim	9 (9.6)	0 (0)	3 (21.8)	12 (9)
Sikh	62 (66)	17 (67.8)	6 (51.9)	85 (65.3)
Caste, n (%)				
General	41 (43.6)	16 (55.7)	5 (39.9)	62 (45.2)
Schedule caste or tribe	36 (38.3)	6 (21.2)	6 (48.3)	48 (36.4)
Other backward class	15 (16)	3 (9.8)	2 (11.8)	20 (14.8)
Other	2 (2.1)	3 (13.3)	0 (0)	5 (3.6)
Ration card, n (%)				
Yes	45 (47.9)	16 (45.7)	5 (35.9)	66 (48.0)
No	49 (52.1)	12 (54.3)	8 (64.1)	69 (52.0)
Parity, n (%)				
1	50 (53.2)	14 (51.6)	8 (60.2)	72 (53.4)
>1	44 (46.8)	14 (48.4)	5 (39.8)	63 (46.6)
Mobile phone ownership, n (%)				
Individual	93 (98.9)	25 (91.6)	9 (80.6)	122 (92.5)
Household	88 (93.6)	28 (100)	13 (100)	134 (99.2)

### Maternal Knowledge of Infant Danger Signs

Despite increases noted across time, maternal knowledge of infant danger signs remained relatively low (Table 3). Of the 12 infant risk factors, the mean number known across arms ranged between 1.85 and 2.31 preintervention and 1.81 and 2.22 postintervention (Table 3 and Multimedia Appendix 1). Being in the synchronous arm was associated with a small but significantly greater increase in the mean number of infant danger signs known when compared to those in the control arm (mean difference 0.87, 95% CI 0.06‐1.69; Table 3). No differences were identified between synchronous versus asynchronous arms or between asynchronous versus control arm participants.

**Table 3.** Comparisons between pre- and postintervention newborn health-related knowledge by intervention arm (n=135).^a^

	Mean (95% CI)			Arm × time parameter (95% CI)		
	Synchronous^b^	Asynchronous^c^	Control	Synchronous vs asynchronous	Synchronous vs control	Asynchronous vs control
Maternal knowledge of infant danger signs (total possible score=12)				0.56 (–0.22 to 1.35)	0.87 (0.06 to 1.69)	0.31 (–0.75 to 1.37)
Preintervention	1.85 (1.61‐2.08)	2.06 (1.47‐2.65)	2.31 (1.19‐3.44)	—^d^	—	—
Postintervention	2.22 (2.00‐2.44)	1.87 (1.32,2.43)	1.81 (1.02‐2.60)	—	—	—
Maternal knowledge of appropriate infant and young child feeding practices (total possible score=9)				–0.75 (–1.96 to 0.45)	0.66 (–2.07 to 3.40)	1.42 (–1.55 to 4.40)
Preintervention	7.56 (7.23‐7.89)	6.48 (5.56‐7.40)	6.84 (5.05‐8.65)	—	—	—
Postintervention	7.89 (7.77‐8.01)	7.56 (6.95‐8.18)	6.51 (4.74‐8.28)	—	—	—

a Full model output for these analyses with corresponding *P* values is presented in Multimedia Appendix 1.

b Combining all participants assigned to weekly group calls.

c Combining those participants assigned to the app and interactive voice response.

d Not applicable.

### Maternal Knowledge of Appropriate Infant and Young Child Feeding Practices

Maternal knowledge of appropriate initiation of varied food groups was high preintervention, and no increase was observed over time (Table 3). Of 9 food groups in total, the mean number of food groups that women reported the correct knowledge for ranged from 6.48 to 7.56 preintervention and 6.51 to 7.89 postintervention. No group differences were identified.

### Infant Health Checkup and Infant Vaccination

Table 4 presents a postintervention comparison of outcomes. Over 50% of participants in the synchronous arm and 40% of participants in the asynchronous arm reported receiving a health checkup for their infants, compared to 28.9% in the control arm. However, no statistically significant difference was noted between arms. Regarding receipt of infant health checkups by a clinical provider, a higher proportion (53.2%) of participants in the synchronous arm reported receiving a health checkup from a clinical provider compared to the other 2 arms. Participants in the synchronous arm had 2.72 times greater odds ratio (OR 2.72, 95% CI 1.02-7.23; *P*<.05) of infant health checkup by a clinical provider compared to the asynchronous arm. There were no statistically significant differences between the synchronous arm and control arm or between the asynchronous arm and control arm. The coverage of all 4 age-appropriate vaccines—BCG (Bacillus Calmette-Guérin), polio, DPT (diphtheria, pertussis, and tetanus), and hepatitis B—was high across all arms, ranging from 89% in the synchronous arm, 84% in the asynchronous arm, and 80.3% in the control arm. No differences were noted in the postintervention between-arms comparison.

**Table 4.** Postintervention comparison of infant health care seeking, vaccination uptake, and infant and young child feeding practices at endline (n=135).

	Synchronous (n=94), n (%)	Asynchronous (n=28), n (%)	Control (n=13), n (%)	Synchronous vs asynchronous, OR^a^ (95% CI)	Synchronous versus control, OR (95% CI)	Asynchronous versus control, OR (95% CI)
Infant health checkup						
Participants who had a postnatal health check for infant within the 6 weeks after giving birth	54 (57.5)	8 (40.9)	2 (28.9)	1.94 (0.71‐5.30)	3.32 (0.64‐17.02)	1.70 (0.25‐11.37)
Health checkup conducted by clinical provider (Ref: no health checkup by a clinical provider)	50 (53.2)	7 (29.5)	2 (24.8)	2.72^b^ (1.02‐7.23)	3.44 (0.69‐17.06)	1.26 (0.20‐7.90)
Mothers whose infants fell sick in the past 3 months	36 (38.3)	7 (33.1)	5 (46.3)	1.25 (0.44‐3.52)	0.71 (0.17‐3.06)	0.57 (0.10‐3.28)
Infants received BCG^c^, polio, DPT^d^, or hepatitis B vaccines						
BCG vaccination	87 (92.6)	23 (96.1)	11 (100)	—^e^	—	—
Polio vaccination	94 (100)	24 (96.6)	10 (93.5)	—	—	—
DPT vaccination	93 (98.9)	24 (100)	11 (100)	—	—	—
Hepatitis B vaccination	92 (97.9)	24 (100)	10 (100)	—	—	—
Received all 4 vaccines (Ref: Received fewer than 4 vaccines)^f^	84 (89)	22 (84)	10 (80.3)	1.59 (0.48‐5.22)	2.06 (0.45‐9.28)	1.28 (0.23‐7.15)
Early initiation of breastfeeding their infant						
Within 1 hour of delivery (Ref: After 1 hour of delivery)	41 (47)	9 (42.1)	5 (56)	1.25 (0.47‐3.29)	0.71 (0.18‐2.79)	0.56 (0.11‐2.85)
Intended length of breastfeeding						
Intend to breastfeed for >24 months (Ref:<24 months)	17 (19.8)	9 (37.6)	2 (14.9)	0.40 (0.15‐1.13)	1.40 (0.25‐7.86)	3.43 (0.50‐23.4)
Mothers who have introduced complementary foods postintervention	87 (92.6)	20 (95.3)	6 (52.2)	0.61 (0.06‐5.50)	11.37^g^ (2.49‐51.88)	18.37^b^ (1.48‐228.20)

a OR: odds ratio.

b These values were statistically significant (*P*<.05).

c BCG: Bacillus Calmette-Guérin.

d DPT: diphtheria, pertussis, and tetanus.

e Not applicable.

f Vaccines include BCG, polio, DPT, and hepatitis B.

g This value was statistically significant (*P*<.001).

### Breastfeeding and Complementary Food Introduction

Initiation of breastfeeding within the first hour after birth was low across all arms (Table 4). The intention to breastfeed for more than 24 months was highest in the asynchronous arm at 37.6%, followed by 19.8% in the synchronous arm and 14.9% in the control arm. No differences in breastfeeding initiation or intention were noted in the postintervention between-arms comparison. A greater proportion of participants in the synchronous arm (92.6%) and the asynchronous arm (95.3%) had introduced complementary foods by 6 months postnatal, compared to the control arm (52.2%).

## Discussion

### Principal Findings

Our pilot study on the preliminary effectiveness of the *MeSSSSage* mHealth education and virtual social support intervention found that synchronous arm assignment had a beneficial impact on participants’ knowledge regarding infant danger signs and odds of obtaining an infant health checkup from a clinical provider when compared to control participants. We also noted no differences by arm in maternal knowledge of appropriate infant and young child feeding practices, number of infant health checkups, and early initiation of breastfeeding. Given the pilot nature of this investigation, including our study design limitations, in conjunction with our previously reported results supporting intervention feasibility and acceptability [51], these findings support continued investigation into the effectiveness of mHealth-based interventions targeting postnatal maternal and newborn health using robust research designs.

Our findings that the *MeSSSSage*’s synchronous arm that included group calls on education and social support increased maternal knowledge of infant danger signs are consistent with existing literature. Previous literature has demonstrated the effectiveness of mHealth interventions in empowering mothers to recognize infant danger signs [28], while interventions layering health education within women’s self-help groups have shown promise in improving maternal knowledge across a range of outcomes [55,56]. Group-based mHealth education and social support interventions can promote health-seeking behaviors through the mechanism of “positive psychological support” among mothers [57]. On the other hand, we found less improvement in participants assigned to asynchronous modes of mHealth intervention, potentially due to unidirectional messaging and lack of social support. Our findings underscore the potential for integrating mHealth delivery into comprehensive interventions that combine both social support and health education, thereby enhancing their impact and effectiveness.

Our study findings noted no difference between pre- and postintervention on maternal knowledge of appropriate infant and young child feeding practices, though scores were high (7 out of 9) at preintervention. It is possible that high baseline levels of maternal knowledge precluded changes over time. A similar lack of effect was also noted in another Indian evaluation of an mHealth intervention for maternal knowledge of infant and child feeding practices [39] and other global studies [37]. Moreover, large-scale evaluations of mHealth interventions in India have failed to observe changes in infant and young child feeding practices [38,39]. Nonetheless, insights from another evaluation in India, including those using innovative approaches like audiovisual tools and interactive messaging, highlight the potential for future mHealth interventions to effectively impact maternal knowledge and practices concerning infant and young child feeding [58].

The synchronous arm of our mHealth intervention demonstrated the greatest impact, particularly in improving infant health checkups by clinical providers. In contrast, our asynchronous intervention arm, comprising solely audio educational messages, showed lower effectiveness. This could be attributed to various factors such as missed calls, insufficient attention to messages, or competing household obligations, resulting in null results for the intervention. Additionally, the absence of impact from the asynchronous modality may also be attributed to instances where participants’ phones were with their husbands during the day, rendering it impossible for them to listen to the IVR messages. Similar challenges were encountered in a study conducted in Punjab, North India, where participants faced connectivity issues and missed messages due to household chores or not having access to their shared phone at the time [50,59]. To address these challenges, future research should identify the optimal timing for sending messages and IVR calls, and devise strategies to enhance engagement and participation in mHealth interventions, such as exploring incentives such as discounted call rates to motivate participants to carefully listen to all messages, as suggested in a study conducted in Afghanistan [60]. Given these challenges, combined effectiveness and implementation designs for mHealth research are likely to be more informative, particularly those that engage qualitative research methods.

The primary objective of our overall study was to assess the feasibility and acceptability of the intervention rather than robustly evaluating its effectiveness; thus, the preliminary effectiveness analysis reported here represents a secondary objective of the study, and the interpretation of these findings should consider the study’s limitations. The sample size was determined for our feasibility and acceptability outcomes, resulting in significant differences in sociodemographic characteristics between the 3 intervention groups at baseline. While weighting techniques were used to address this imbalance, more robust experimental designs with a larger sample size will allow for a better assessment of effectiveness and potential mechanisms of impact. We also noted differential retention across the study by intervention group, which may have influenced our findings. Furthermore, our study design also limited our ability to assess whether intervention effectiveness differed by sociodemographic characteristics, and considering the important influence of social and structural factors on women of reproductive age and their influence on perinatal health, future research should focus on understanding the potential of interventions to specifically mitigate health disparities.

### Conclusion

The postnatal period presents a critical opportunity to engage new mothers in enhancing their knowledge and practices concerning infant and young child feeding, infant health checkups and care-seeking behaviors, child vaccinations, and providing social support. Our pilot study on the *MeSSSSage* mHealth education and virtual social support intervention delivered mostly in the postnatal period yielded modest results and offered promising insights into its preliminary effectiveness. Such interventions, integrating mHealth-based education and communication with social support, hold significant promise but warrant further exploration to optimize their impact. With smartphones and social media platforms like WhatsApp increasingly prevalent even in low-resource settings, there is an urgent need for more rigorous experimental research to comprehensively evaluate the impact of mHealth interventions and their underlying mechanisms. Our team is currently conducting a fully powered randomized controlled trial to examine the effectiveness and potential mechanisms of impact of an mHealth educational and social support intervention for perinatal women across multiple sites in India, with results expected in the coming years.

## Supplementary material

10.2196/65581 Multimedia Appendix 1 Model output for the analyses.

## Abbreviations

ASHA: Accredited Social Health Activist
BCG: Bacillus Calmette-Guérin
COM-B: Capability, Opportunity, and Motivation Behavior
DPT: diphtheria, pertussis, and tetanus
IVR: interactive voice response
LMIC: low- and middle-income country
*MeSSSSage*: *Maa Shishu Swasthya Sahayak Samooh* (which means maternal and child health support group)
mHealth: mobile health
OR: odds ratio
REDCap: Research Electronic Data Capture
