# Supplementary material for: Preliminary Effectiveness of a Postnatal mHealth and Virtual Social Support Intervention on Newborn and Infant Health and Feeding Practices in Punjab, India: Quasi-Experimental Pre-Post Pilot Study
Source: JMIR Pediatr Parent. 2025 Jun 27;8:e65581. doi: 10.2196/65581 (PMC12227177; doi:10.2196/65581)
Supplement: Multimedia Appendix 1 [file pediatrics-v8-e65581-s001.docx]

**Model output for the analyses**

|  | **Synchronous vs asynchronous arm** | | | | | | **Synchronous vs control arm** | | | | | | **Asynchronous vs control arm** | | | | | |
| --- | --- | --- | --- | --- | --- | --- | --- | --- | --- | --- | --- | --- | --- | --- | --- | --- | --- | --- |
|  | **Arm parameter (95% CI)** | ***P* value** | **Time parameter (95% CI)** | ***P* value** | **Arm*Time parameter (95% CI)** | ***P* value** | **Arm parameter (95% CI)** | ***P* value** | **Time parameter (95% CI)** | ***P* value** | **Arm*Time parameter (95% CI)** | ***P* value** | **Arm parameter (95% CI)** | ***P* value** | **Time parameter (95% CI)** | ***P* value** | **Arm*Time Parameter (95% CI)** | ***P* value** |
| Maternal knowledge of infant danger signs | –0.20 (–0.82 to 0.42) | .52 | –0.19 (–0.91 to 0.53) | .61 | 0.56 (–0.22 to 1.35) | .16 | –0.44 (–1.54 to 0.66) | .43 | –0.51 (–1.30 to 0.25) | .19 | 0.87* (0.06 to 1.69) | .03 | –0.25 (–1.52 to 1.01) | .69 | –0.50 (–1.27 to 0.26) | .2 | 0.31 (–0.75 to 1.37) | .56 |
| Maternal knowledge on infant and young child feeding practices | 1.08* (0.11 to 2.05) | .03 | 1.08 (–0.07 to 2.24) | .06 | –0.75 (–1.96 to 0.45) | .22 | 0.71 (–1.10 to 2.53) | .44 | –0.33 (–3.05 to 2.38) | .81 | 0.66 (–2.07 to 3.40) | .63 | –0.37 (–2.39 to 1.66) | .72 | –0.33 (–3.07 to 2.40) | .35 | 1.42 (–1.55 to 4.40) | .35 |

Robust chi in parentheses.

****P*<.001, ***P*<.01, **P*<.05.
